# Synthesis of C3/C1-Substituted Tetrahydroisoquinolines

**DOI:** 10.3390/molecules200814902

**Published:** 2015-08-14

**Authors:** Mohamed Mihoubi, Nicola Micale, Angela Scala, Raoudha Mezghani Jarraya, Amira Bouaziz, Tanja Schirmeister, Francesco Risitano, Anna Piperno, Giovanni Grassi

**Affiliations:** 1 Laboratoire de Chimie des Substances Naturelles UR/11-ES-74, Faculté des Sciences de Sfax, Université de Sfax, Route de l’aeroport, BP 1171, 3000 Sfax, Tunisia; E-Mails: mohamed.mihoubi@hotmail.fr (M.M.); raoudhajarraya@yahoo.fr (R.M.J.); bouaziz.amira@yahoo.fr (A.B.); 2 Dipartimento di Scienze del Farmaco e Prodotti per la Salute, Università degli Studi di Messina, Viale Annunziata, 98168 Messina, Italy; E-Mail: nmicale@unime.it; 3 Dipartimento di Scienze Chimiche, Università di Messina, Viale Ferdinando Stagno D’Alcontres 31, 98166 Messina, Italy; E-Mails: ascala@unime.it (A.S.); frisitano@unime.it (F.R.); ggrassi@unime.it (G.G.); 4 Institute of Pharmacy and Biochemistry, University of Mainz, Staudinger Weg 5, D 55099 Mainz, Germany; E-Mail: schirmei@uni-mainz.de

**Keywords:** Bischler-Napieralski condensation, *N*-methylisosalsoline, nitroalkene, proteasome, tetrahydroisoquinoline

## Abstract

A broad biological screening of the natural alkaloid *N*-methylisosalsoline (**2**) extracted from *Hammada* *scoparia* leaves against a panel of human and parasitic proteases revealed an interesting activity profile of **2** towards human 20S proteasome. This outcome suggests that the 1,2,3,4-tetrahydroisoquinoline skeleton may be exploited as a template for the development of novel anticancer agents. In this article, we report the synthesis and chemical characterization of a new series of isosalsoline-type alkaloids (**10**–**11**) with variations at N2 and C3 positions with respect to the natural Compound **2**, obtained by a synthetic strategy that involves the Bischler-Napieralski cyclization. The substrate for the condensation to the tetrahydroisoquinoline system, *i.e*., a functionalized β-arylethyl amine, was obtained through an original double reduction of nitroalkene. The synthetic strategy can be directed to the construction of highly substituted and functionalized 1,2,3,4-tetrahydroisoquinolines.

## 1. Introduction

The 1,2,3,4-tetrahydroisoquinoline (THIQ) skeleton is an important structural motif commonly encountered in naturally-occurring alkaloids of vegetal origin with interesting biological/pharmacological properties, including inhibition of cell proliferation/anticancer activity [1], multiple cardiovascular activities related to different mechanisms of action [2,3,4], anti-inflammatory [5], antimicrobial [6,7], antiplasmodial activities [8,9] and acetylcholinesterase inhibitory effects [10,11]. A variety of THIQ derivatives has been found also in mammals, especially in human brain, suggesting a role of these molecules in neurons under physiological and pathological conditions [12]. 

The structural diversity and the wide range of biological activities of THIQs have long attracted the attention of synthetic chemists, and several druggable semi-synthetic and synthetic THIQ-related compounds have been obtained in the last decade with simple and efficient methods [13,14,15,16,17,18,19,20,21,22].

The majority of the natural THIQ alkaloids endowed with drug-like properties have been isolated from plants of the genus *Corydalis* (Papaveraceae), and traditionally, some *Corydalis* species have long been used for the treatment of different aliments in Eastern Asian countries [23]. 

**Figure 1:**
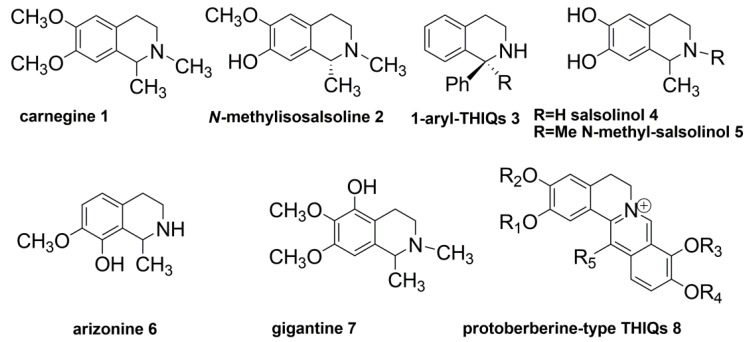
Structure of some natural and synthetic 1,2,3,4-tetrahydroisoquinoline (THIQ) alkaloids.

Recently, two THIQ alkaloids, carnegine (**1**) and *N*-methylisosalsoline (**2**) (Figure 1), have been isolated from leaf extracts of *Hammada* *scoparia* (Chenopodiaceae). Both compounds showed molluscicidal activity against *Galba truncatula*, the principal intermediate host of the parasitic trematode *Fasciola hepatica*, the causative agent of fasciolosis in Southern Tunisia [24]. *N*-methylisosalsoline (**2**) showed the highest molluscicidal activity, and therefore, it was selected for further investigations. 

As part of an ongoing program of targeting small molecules of natural and synthetic origin, **2** was tested against a panel of human and parasitic proteases at our disposal, including cathepsin-B and -L, human 20S proteasome, recombinant *Leishmania mexicana* cysteine protease CPB2.8, Rhodesain and Dengue virus NS2B/NS3 protease. From this screening, Compound **2** turned out to be active against human 20S proteasome, suggesting a potential as a lead structure for the development of anticancer agents. The screening at 20 µM produced ~40% of the inhibition of proteasome β5-subunits (chymotrypsin-like activity), ~20% of the inhibition of β2-subunits (trypsin-like activity) and no inhibition towards β1-subunits (caspase-like activity). Although the antiproteasomal activity of **2** can be classified as “fair”, its inhibition profile can be considered “ideal” for a drug candidate due to the fact that the β5-subunits are mainly involved in protein turnover (thus representing the primary targets for the development of efficacious anticancer agents) and that the co-inhibition of the β5-subunits with either the β1- or the β2-subunits produces the maximal antitumor response [25,26,27]. Conversely, the inhibition of all three proteasome proteolytic subunits (β1, β2 and β5) may result in cytotoxicity [28]. Moreover, **2** did not show any inhibitory activity in the cross-reactivity test against bovine pancreatic α-chymotrypsin. This interesting outcome prompted us to devise a new synthetic method to obtain THIQ derivatives with variations at 2–3 positions compared to **2** in the attempt to optimize the biological profile of our lead structure toward 20S proteasome and to provide at the same time a simple and alternative strategy for the construction of the THIQ ring system. 

The other positions of the THIQ scaffold were purposely kept intact, as it is known from the literature that small modifications of this framework may lead to miscellaneous activities. For instance, the 1-aryl-substituted THIQs (**3**) usually interfere with glutamate neurotransmission and may have a major significance as neuroprotective agents [29]. Salsolinol (**4**), 1-methyl-6,7-dihydroxy-THIQ and its *N*-methyl-derivative (**5**) have been identified as endogenous neurotoxins to dopamine neurons [12]. A diverse substitution pattern with respect to **2** on the phenyl ring as in the Cactaceae alkaloids arizonine (**6**) and gigantine (**7**) may cause unspecific toxic effects involving the central nervous system [30]. More complex structures, such as protoberberine-type alkaloids (**8**), display high antiviral activities, but they are also very active against acetylcholinesterase [23].

For the formation of the THIQ ring system, the Pictet-Spengler and Bischler-Napieralski condensations are the most efficient methods employed in drug discovery, although their lack of substrate generality has limited the access to C3- and C4-substituted THIQs [31]. 

**Figure 2:**
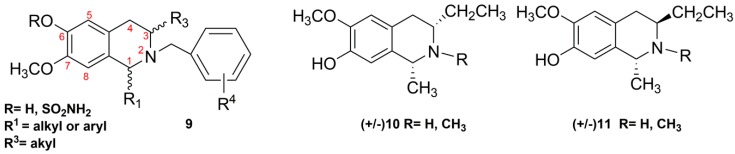
Structures of the known C3-/C1-substituted THIQs (**9**) endowed with antiproliferative activities and the C3/C1-substituted (**10**–**11**) THIQs reported herein.

The presence of phenolic groups at the C6 and/or C7 position of the THIQ skeleton is often imperative for the maintenance of the biological properties. For example, the antitubulin and antiproliferative activities of C3/C1-substituted THIQs **9** (Figure 2) depend on the pharmacophore group at C6 (as free phenol or sulfamate group) [32,33,34]. Therefore, procedures of the benzyl protection/deprotection of phenolic groups have been employed in the literature showing in many cases some incompatibility with the other functional groups present on the THIQ skeleton [33].

Herein, we report direct access to C3/C1 THIQs Derivatives **10**–**11** (Figure 2) by Bischler-Napieralski cyclization of the appropriately-functionalized β-arylethylamine intermediate, which was obtained in turn by the reduction and acylation of the corresponding nitroalkene*.* Our synthetic method can be applied to the synthesis of diversely-substituted (C1, N2, C3, C6, C7) THIQs avoiding the benzyl protection/deprotection procedures. Considering that THIQs **9** contain substituents at N2/C3 or N2/C1, the synthesis of Compounds **10**–**11** herein reported describes for the first time the preparation of C1/C3-disubstituted or C1/N2/C3-trisubstituted isosalsoline-type alkaloids.

## 2. Results and Discussion

### 2.1. Evaluation of N-Methylisosalsoline *(**2**)* against Human and Parasitic Proteases

Compound **2** underwent a preliminary screening for the above-mentioned panel of human and parasitic proteases activity at 20 µM, using an equivalent volume of dimethyl sulfoxide (DMSO) as a negative control and the appropriate fluorogenic substrate [e.g., Suc-Leu-Leu-Val-Tyr-AMC (AMC stands for 7-amino-4-methycoumarim)] for the proteasomal chymotrypsin-like (ChT-L) activity; Boc-Leu-Arg-Arg-AMC for the trypsin-like (T-L) activity; Z-Leu-Leu-Glu-AMC for the post-glutamyl peptide hydrolyzing (PGPH) activity (see the Experimental Section for details about all proteases). Compound **2** did not show any inhibitory activity in most cases, with the exception of recombinant *Leishmania mexicana* cysteine protease CPB2.8 (~14%) and of the already discussed β5-subunits of human 20S proteasome (~40%). However, since **2** did not inhibit more than 50% any of the enzymatic activities, continuous assays were not performed according to our standard policy.

### 2.2. Synthesis of C3/C1-Substituted THIQs *(**10**–**11**)*

**Scheme 1:**
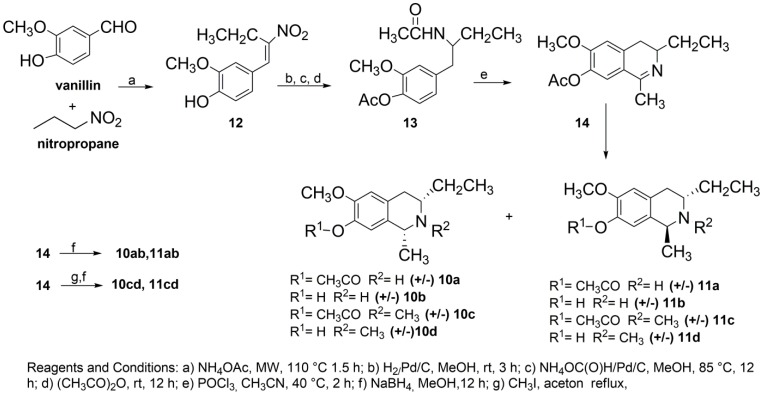
Synthesis of isosalsoline-type Alkaloids **10**–**11**.

The approach used to generate the C3/C1-substituted THIQ core was based on the preparation of 4-(2-acetamidobutyl)-2-methoxyphenyl acetate **13** and the subsequent Bischler-Napieralski cyclodehydration (Scheme 1). Our synthesis began with the Henry condensation reaction between vanillin and nitropropane, performed in the presence of a catalytic amount of ammonium acetate, in a solventless system and under microwave irradiation (Scheme 1). The condensation was carried out without the protection of the phenolic group of vanillin and afforded the nitroalkene **12** in 70% yield.

The key step in the proposed synthetic approach is the conversion of nitroalkene **12** into the *N*-acetyl-β-arylethyl amine derivative **13**. To achieve the reduction of the α,β-unsaturated nitro group, common literature methods [35] entail the use of LiAlH_4_ and lead to very low yield reactions. The use of anhydrous ammonium formate in conjunction with Pd-C catalyst gave the oxime derivatives as the main products [35]. Good results in our case were obtained by a “step by step” selective reduction procedure based on the catalytic hydrogenation of the alkene functionality at room temperature (TLC monitoring) followed by the reduction of the aliphatic nitro group by adding to the reaction mixture ammonium formate and raising the temperature to reflux. The crude product of this double reduction was then treated with acetic anhydride to obtain, after chromatographic purification, the *N*-acetyl-β-arylethyl amine derivative **13** in 40% overall yield. **13** underwent standard cyclodehydration with POCl_3_ to give the dihydroisoquinoline derivative **14** in a quantitative yield.

The reduction of the imine moiety of **14** with NaBH_4_ afforded the corresponding THIQs **10a**,**b** and **11a**,**b** in a 70% yield. The dihydroisoquinoline intermediate **14** was also *N*-methylated to dihydroisoquinolinium salt prior to the reduction with NaBH_4_ to obtain the THIQs **10c,d** and **11c,d**. Compounds **10** and **11** were obtained as a mixture of acetylated (**10a**, **10c**, **11a**, **11c**) and non-acetylated derivatives (**10b**, **10d**, **11b**, **11d**), which were eventually separated by flash chromatography using ethyl acetate/acetonitrile/methanol (7/1.5/1.5) as the eluent.

The structure of compounds **10a**–**d** and **11a**–**d** was determined on the basis of analytical and spectroscopic data. Their relative *cis*/*trans* configurations were assigned on the basis of NOE experiments by correlation of the H1 and H3 protons: *i.e.*, for compound **10b**, the irradiation of the H-1 proton at 4.12 ppm (d) resulted in a signal enhancement for H-3 (m, 2.83 ppm), for H3ʹa (m, 1.45 ppm) and for aromatic protons. These observations support the compound **10a** *cis* relationship for methyl and ethyl groups at C1 and C3 position. 

## 3. Experimental Section 

### 3.1. General Information 

Melting points were determined on a Kofler melting apparatus and are uncorrected. ^1^H- and ^13^C-NMR spectra were obtained on a Varian 500-MHz spectrometer (Palo Alto, CA, USA). The chemical shifts (δ) and coupling constants (*J*) are expressed in ppm and Hertz, respectively. Microanalyses and mass spectrometry analyses were carried out on a Carlo Erba EA 1102 and on a 3200 QTRAP (Applied Biosystem SCIEX, Foster City, CA USA), respectively. All solvents and reagents were obtained from commercial sources and purified before use if necessary. Merck (Darmstadt, Germany) Kieselgel 60F254 plates were used for TLC and Merck Silica gel 60 (0.063–0.100 mm) for column chromatography.

Human 20S proteasome was obtained from Biomol GmbH, Hamburg, Germany. The three distinct proteolytic activities of the 20S proteasome were measured by monitoring the hydrolysis of the peptidyl 7-amino-4-methyl coumarin substrates (all obtained from Bachem) Suc-Leu-Leu-Val-Tyr-AMC, Boc-Leu-Arg-Arg-AMC and Cbz-Leu-Leu-Glu-AMC for ChT-L, T-L and PGPH activity of the enzyme, respectively. Fluorescence of the product AMC of the substrates’ hydrolyzes was measured using an Infinite 200 PRO microplate reader (Tecan, Männedorf, Switzerland) at 30 °C with a 380-nm excitation filter and a 460-nm emission filter. The preliminary screening for the inhibition of the three proteolytic activities of the 20S proteasome was performed at 20 mM inhibitor concentrations using an equivalent amount of DMSO as a negative control. Recombinant enzymes were expressed as previously described [36,37]. Cathepsins B and L were purchased from Calbiochem, and the assays were performed as described previously [38] Cbz-Phe-Arg-AMC was used as the substrate (80 µM for cathepsin-B; 5 µM for cathepsin-L). The proteases NS2B-NS3 of DENV2 and DENV3 were recombinantly expressed as described previously [39].

### 3.2. Extraction of N-Methylisosalsoline ***2***

*N*-Methylisosalsoline **2** was obtained from *Hammada scoparia* leaves according to a literature method. Briefly, air-dried leaves of *Hammada scoparia* were extracted at room temperature during 48 h with a mixture (EtOH/H_2_O, 1/9). After filtration, the ethanol was removed under reduced pressure, and the remaining aqueous phase was acidified with HCl (pH *=* 3) and then defatted by extraction with CH_2_Cl_2_. NH_4_OH solution was added to aqueous phases (pH *=* 10) and immediately extracted with CH_2_Cl_2_. The organic extracts were concentrated to yield a reddish-brown residue (total alkaloids). The residue (5 g) was separated, on column chromatography over silica gel using a gradient of dichloromethane-methanol to give *N*-methylisosalsoline (545 mg) as white rosette crystals. The data of the ^1^H-NMR spectra matched those reported in the literature [40]. 

### 3.3. Evaluation of ***2*** against Human and Parasitic Proteases

Alkaloid **2** was tested against a panel of human and parasitic proteases, including cathepsin-B and -L, human 20S proteasome, recombinant *Leishmania mexicana* cysteine protease CPB2.8, rhodesain and Dengue virus NS2B/NS3 protease.

#### 3.3.1. Assaying the Chymotryptic Activity of the 20S Proteasome

Human 20S proteasome was incubated at 30 °C at a final concentration of 0.004 mg∙mL^−1^ with test compound present at 20 µM. The reaction buffer consisted of 50 mM Tris pH 7.5, 10 mM NaCl, 25 mM KCl, 1 mM MgCl_2_, 0.03% SDS and 5% DMSO. Product release from substrate hydrolysis (75 mM) was monitored continuously over a period of 10 min.

#### 3.3.2. Assaying the Tryptic Activity of the 20S Proteasome

Human 20S proteasome was incubated at 30 °C at a final concentration of 0.0025 mg∙mL^−1^ with the test compound present at 20 µM. The reaction buffer consisted of 50 mM Tris buffer pH 7.4, 50 mM NaCl, 0.5 mM EDTA, 0.03% SDS and 7.5% DMSO. Product release from substrate hydrolysis (85 mM) was monitored continuously over a period of 10 min.

#### 3.3.3. Assaying the Post-Glutamyl Peptide Hydrolyzing Activity of the 20S Proteasome

Human 20S proteasome was incubated at 30 °C at a final concentration of 0.004 mg∙mL^−1^ with the test compound present at variable concentrations. The reaction buffer consisted of 50 mM Tris buffer pH 7.5 containing 25 mM KCl, 10 mM NaCl, 1 mM MgCl_2_, 0.03% SDS, 5% DMSO. Product release from substrate hydrolysis (80 mM) was monitored continuously over a period of 10 min.

#### 3.3.4. Assay for Bovine Pancreatic α-Chymotrypsin Inhibition

The enzyme (250 mg∙mL^−1^) was incubated at 20 °C with the test compound. The reaction buffer consisted of 50 mM Tris buffer pH 8.0 containing 100 mM NaCl, 5 mM EDTA and 7.5% DMSO. Product release from substrate hydrolysis (75 mM final concentration, Suc-Leu-Leu-Val-Tyr-AMC from Bachem) was determined over a period of 10 min.

#### 3.3.5. Assay for Rhodesain Inhibition

Product release from substrate hydrolysis (Cbz-Phe-Arg-AMC; 10 µM) was determined continuously over a period of 10 min. The enzyme was incubated at 20 °C with the test compound in a 50 mM sodium acetate buffer (pH 5.5) containing 10 mM 1,4-di-thiothreitol (DTT), 5 mM EDTA and 200 mM NaCl.

#### 3.3.6. Assay for Leishmania Mexicana Cysteine Protease CPB2.8

Product release from substrate hydrolysis (Cbz-Phe-Arg-AMC, 10 µM) was determined continuously over a period of 10 min. The enzyme was incubated at 20 °C with the test compound in a 50 mM sodium acetate buffer (pH 6.5) containing 5 mM 1,4-dithiothreitol (DTT) and 5 mM EDTA.

#### 3.3.7. Assay for Dengue Virus NS2B/NS3 Protease 

Product release from substrate hydrolysis (Boc-Gly-Arg-Arg-AMC, 100 µM) was determined continuously over a period of 10 min. The enzyme was incubated at 25 °C with the test compound in a 50 mM Tris buffer (pH 9.0) containing 1 mM CHAPS and 20% glycerol (*v*/*v*).

### 3.4. Synthesis of (E)-2-Methoxy-4-(2-nitrobut-1-enyl)phenol ***12***

A catalytic amount of ammonium acetate (192 mg, 2.5 mmol) was added to a mixture of vanillin (1.5 g, 10 mmol) and nitropropane (5 mL, 50 mmol). The mixture was irradiated by microwaves for 90 min at 110 °C. The reaction mixture was washed with water and extracted with ethyl acetate; the organic phases were evaporated under reduced pressure, and the residue was purified by silica gel column chromatography (hexane/EtOAc, 8:2) to obtain 1.56 g of **12** as a yellow solid crystal. 

*2-Methoxy-4-(2-nitrobut-1-enyl)phenol* (**12**); 70% yield, mp *=* 42 °C. ^1^H-NMR (500 MHz, CDCl_3_): δ *=* 1.30 (t, *J* *=* 7.4 Hz, 3H), 2.80 (q, *J* *=* 7.4 Hz, 2H), 3.93 (s, 3H), 5.91 (s, 1H), 6.94 (d, *J* *=* 2.0 Hz, 2H), 6.99 (d, *J* *=* 8.4 Hz, 1H), 7.30 (dd, *J* *=* 2.0 and 7.4 Hz, 1H ), 7.99 (s, 1H) ;^13^C-NMR (125 MHz, CDCl_3_): δ *=* 12.3, 21.5, 55.9, 112.1, 115.7, 124.0, 125.6, 133.9, 147.5, 147.9, 151.7; ESI-HRMS: calcd. for C_11_H_13_NO_4_ + H 224.0924, found 224.0925.

### 3.5. Synthesis of 4-(2-Acetamidobutyl)-2-methoxyphenyl Acetate ***13***

To a solution of Compound **12** (1.15 g, 5 mmol) in dry methanol (50 mL), under argon, 120 mg of Pd/C were added. The reaction mixture was subjected to a hydrogen flux for two hours at room temperature. Anhydrous ammonium formate (1.45 g, 23 mmol) was added, and the reaction was refluxed for four hours. Then, a second portion of ammonium formate (1.45 g, 1 mmol) was added, and the reaction was refluxed for four hours. The reaction mixture was filtrated, and the solution was evaporated to dryness. The residue was treated with acetic anhydride for 12 h and then evaporated. The residue was triturated with water, extracted with CH_2_Cl_2_ and dried over Na_2_SO_4_. The organic layers were evaporated, and the residue was purified by silica gel column chromatography (hexane/EtOAc, 1:1) to obtain 560 mg of **13** as oil.

*4-(2-Acetamidobutyl)-2-methoxyphenyl acetate* (**13**); 40% yield. ^1^H-NMR (500 MHz, CDCl_3_): δ *=* 0.92 (t, *J =* 7.3 Hz, 3H), 1.36 (m, 1H), 1.59 (m, 1H), 1.98 (s, 3H), 2.30 (s, 3H), 2.78 (m, 2H), 3.81 (s, 3H), 4.10 (m, 1H), 5.66 (bs, 1H, NH), 6.72 (dd, *J =* 2.0 and 7.9 Hz, 1H), 6.79 (d, *J =* 2.0, 1H ), 6.93 (d, *J =* 7.9 Hz, 1H); ^13^C-NMR (125 MHz, CDCl3): δ *=* 10.5, 20.9, 23.1, 26.3, 51.9, 58.0, 113.8, 121.7, 122.0, 127.4, 138.2, 151.3, 169.8; ESI-HRMS: calcd. for C_15_H_21_NO_4_ + H 280.1550, found 280.1549.

### 3.6. Synthesis of 3-Ethyl-6-methoxy-1-methyl-3,4-dihydroisoquinolin-7-yl Acetate ***14***

A solution of 4-(2-acetamidobutyl)-2-methoxyphenyl acetate **13** (140 mg, 0.5 mmol) in dry acetonitrile (15 mL) was treated with POCl_3_ (0.23 mL, 2.5 mmol, 383 mg,) and refluxed for 2 h under nitrogen atmosphere. The reaction mixture was evaporated to dryness; the residue was dissolved in H_2_O (10 mL), basified to pH *=* 9 and extracted with CH_2_Cl_2_. The organic phases were dried over anhydrous NaSO_4_, filtered and evaporated to dryness. The imine **14** was directly used in the next reactions. 

*3-Ethyl-6-methoxy-1-methyl-3,4-dihydroisoquinolin-7-yl acetate* (**14**). ^1^H-NMR (500 MHz, CDCl_3_): δ *=* 1.03 (t, *J =* 7.3 Hz, 3H), 1.63 (ddd, *J =* 7.3, 7.8 and 13.3 Hz, 1H), 1.85 (ddd, *J =* 6.2, 7.3 and 13.3 Hz, 1H), 2.31 (s, 3H), 2.37 (s, 3H), 2.50 (dd, *J =* 12.7 and 16.2 Hz, 1H), 2.75 (dd, *J =* 5.4 and 16.2 Hz, 1H), 3.61 (dddd, *J =* 5.4, 6.2, 7.8 and 12.7, 1H), 3.86 (s, 3H), 6.76 (s, 1H), 7.17 (s, 1H); ^13^C-NMR (125 MHz, CDCl3): δ *=* 10.7, 20.6, 22.7, 30.8, 30.9, 56.0, 56.1, 111.4, 111.5, 120.6, 136.9, 138.2, 167.2, 169.0. ESI-HRMS: calcd. for C_15_H_19_NO_3_ + H 262.1444, found 262.1444. 

### 3.7. Synthesis of ***10a**,**b*** and ***11a**,**b***

NaBH_4_ (65 mg, 1.71 mmol) was added to a solution of Imine **14** (150 mg, 0.57 mmol) in 30 mL of methanol. The reaction mixture was stirred at room temperature for 12 h; then, solvent was removed under reduced pressure, and the residue was triturated with chloroform and ethyl acetate. The collected organic phases were evaporated to dryness, and the residue was purified by silica gel column chromatography using ethyl acetate/MeOH/CH_3_CN (7:1.5:1.5) to obtain two mixtures of THIQ **10a**/**11a** and **10b**/**11b** in a 70% global yield as a white oil. 

*3-Ethyl-6-methoxy-1-methyl-1,2,3,4-tetrahydroisoquinolin-7-yl acetate* (**10a** and **11a**): Isomer **10a** ^1^H-NMR (500 MHz, CDCl_3_): δ *=* 1.02 (t, 3H, *J =* 7.3 Hz), 1.46 (d, 3H, *J =* 6.3 Hz), 1.63–165 (m, 2H), 2.30 (s, 3H), 2.55 (m,1H), 2.70 (m, 1H), 2.95 (m,1H), 3.80 (s 3H), 4.10 (q, 1H, *J =* 6.3 Hz), 6.66 (s,1H), 6.84 (s,1H). Isomer **11a** ^1^H-NMR (500 MHz, CDCl_3_): δ *=* 1.01 (t, 3H, *J =* 7.1Hz), 1.43 ( d, 3H, *J =* 6.2 Hz), 1.45 (m, 1H), 1.60 (m, 1H), 2.31 (s, 3H), 2.55 (m, 1H), 2.70 (m, 1H), 2.83 (m,1H), 3.78 (s 3H), 4.12 (q, 1H, *J =* 6.2 Hz), 6.69 (s,1H), 6.85 (s, 1H). ^13^C-NMR of **10a** and **11a** mixture (125 MHz, CDCl_3_) δ *=* 10.3, 20.6, 22.0, 29.3, 29.5, 35.3, 35.8, 52.0,52.3, 55.1, 55.4, 110.9, 111.3, 112.6, 119.4, 125.1, 132.3, 132.4, 135.5, 137.8, 143.9, 145.2, 149.1, 169.3. ESI-HRMS: calcd. for C_15_H_21_NO_3_ + H 263.1521, found 263.1419. 

*3-Ethyl-6-methoxy-1-methyl-1,2,3,4-tetrahydroisoquinolin-7-ol* (**10b** and **11b**). Isomer **10b** ^1^H-NMR (500 MHz, CDCl_3_): δ *=* 1.02 (t, 3H, *J =* 7.3 Hz), 1.46 (d, 3H, *J =* 6.3 Hz), 1.50–1.65 (m, 2H), 2.55 (m,1H), 2.75 (m, 1H), 2.90 (m,1H), 3.85 (s 3H), 4.10 (q, 1H, *J =* 6.3 Hz), 6.55 (s,1H), 6.70 (s,1H). Isomer **10b**: ^1^H-NMR (500 MHz, CDCl_3_): δ *=* 1.02 (t, 3H, *J =* 7.3 Hz), 1.50 (d, 3H, *J =* 6.3 Hz), 1.65–1.70 (m, 2H), 2.55 (m, 1H), 2.75 (m, 1H), 3.05 (m, 1H), 3.86 (s, 3H), 4.15 (q, 1H, *J =* 6.3 Hz), 6.55 (s,1H), 6.60 (s, 1H). ^13^C-NMR of **10b** and **11b** mixture (125 MHz, CDCl3) δ *=* 10.3, 22.3, 29.6, 29.5, 35.2, 35.8, 52.0, 52.3, 55.1, 55.8, 111.0, 111.3, 112.5, 119.4, 126.0, 132.3, 132.4, 135.5, 137.8, 143.9, 145.2. ESI-HRMS: calcd. for C_13_H_19_NO_2_ + H 222.1495, found 222.1497.

### 3.8. Synthesis of ***10c**,**d*** and ***11c**,**d***

A solution of Imine **14** (153 mg, 0.58 mmol) in acetone (30 mL) was treated with CH_3_I (1.26 mL, 2.9 mmol). The reaction was stirred at reflux overnight; then, the solvent was evaporated to dryness and the residue was dissolved in methanol (30 mL) and was treated with NaBH_4_ (35 mg, 1.74 mmol). The reaction mixture was stirred at room temperature for 12 h, and then, the solvent was removed under reduced pressure. The residue was triturated with chloroform and ethyl acetate, and the collected organic phases were evaporated to dryness. The residue was purified by silica gel column chromatography using ethyl acetate/MeOH/CH_3_CN (7:1.5:1.5) to obtain THIQ **10c**,**d** and **11c**,**d** in an 80% yield as a white oil. 

*3-Ethyl-6-methoxy-1,2-dimethyl-1,2,3,4-tetrahydroisoquinolin-7-yl acetate* (**10c** and **11c**). Isomer **10c** ^1^H-NMR (500 MHz, CDCl_3_): δ *=* 0.98 (t, 3H, *J =* 5.2 Hz), 1.37 (d, 3H, *J =* 5.0 Hz), 1.45 (m, 1H), 1.63 (m, 1H), 2.30 (s, 3H), 2.37 (s, 3H), 2.65 (dd, 1H, *J =* 6.55 and 13.1Hz), 2.68 (dd, 1H, *J =* 1.5 and 13.1 Hz), 2.99 (m,1H), 3.72 (q, 1H, *J =* 5.0 Hz), 3.79 (s 3H), 6.65 (s, 1H), 6.68 (s, 1H).Isomer **11c** 1H-NMR (500 MHz, CDCl_3_): δ *=* 0.98 (t, 3H, *J =* 5.2 Hz), 1.46 ( d, 3H, *J =* 5.0 Hz), 1.46 (m, 1H), 1.71 (m, 1H), 2.20 (s, 3H), 2.30 (s, 3H), 2.65 (m, 1H), 2.66 (m,1H), 2.69 (m,1H), 3.80 (s 3H), 3.85 (q, 1H, *J =* 5.0 Hz), 6.65 (s, 1H), 6.80 (s, 1H). ^13^C-NMR of **10c** and **11c** mixture (125 MHz, CDCl_3_) δ *=* 10.6, 11.1, 20.6, 21.6, 21.7, 24.6, 25.1, 36.3, 53.9, 54.1, 55.7, 55.9, 58.3, 58.4, 112.1, 112.3, 112.4, 121.2, 121.3, 131.2, 132.0, 137.9, 167.2, 169.3. ESI-HRMS: calcd. for C_16_H_23_NO_3_ + H 278.1757, found 278.1759. 

*3-Ethyl-6-methoxy-1,2-dimethyl-1,2,3,4-tetrahydroisoquinolin-7-ol* (**10d** and **11d**). Isomer **10d** ^1^H-NMR (500 MHz, CDCl_3_): δ *=* 1.01 (t, 3H, *J =* 5.5 Hz), 1.49 ( d, 3H, *J =* 6.Hz), 1.55–160 (m, 2H), 2.40 (s, 3H), 2.55 (m, 1H), 2.66 (m,1H), 3.05 (m,1H), 3.80 (s, 3H) 4.12 (q, 1H, *J =* 6.0 Hz), 6.55 (s, 1H), 6.66 (s,1H). Isomer **11d** ^1^H-NMR (500 MHz, CDCl_3_): δ *=* 1.02 (t, 3H, *J =* 5.5 Hz), 1.53 (d, 3H, *J =* 6.Hz), 1.55–160 (m, 2H), 2.30 (s, 3H), 2.55 (m, 1H), 2.66 (m, 1H), 3.0 (m, 1H), 3.81 (s, 3H), 3.82 (q, 1H, *J =* 6.0 Hz), 6.57 (s,1H), 6.70 (s,1H). ^13^C-NMR of **10d** and **11d** mixture (125 MHz, CDCl_3_) δ *=* 10.5, 10.8, 20.9, 22.7, 24.3, 25.5, 37.4, 35.3, 54.8, 55.9, 61.9, 110.4, 110.7, 112.2, 112.2, 121.3, 146.0. ESI-HRMS: calcd. for C_14_H_21_NO_2_ + H 236.1851, found 236.1850. 

## 4. Conclusions

The interesting activity profile towards human 20S proteasome highlighted by the natural alkaloid *N*-methylisosalsoline aroused our interest in the research of an alternative synthetic strategy for the construction of new isosalsoline-type alkaloids with variations at the N2 and C3 positions with respect to the natural compound. We proposed an efficient synthetic method, hitherto not described in the literature and applicable to the construction of the THIQ scaffold, which involves the Bischler–Napieralski cyclization of a functionalized *N*-acetyl-β-arylethyl amine obtained through an original double reduction of the corresponding nitroalkene. The biological evaluation of the newly-synthesized compounds (**10**,**11**) is in progress and will be reported at a later date.
